# Patterns of the left thalamus embedding into the connectome associated with reading skills in children with reading disabilities

**DOI:** 10.1162/netn_a_00414

**Published:** 2024-12-10

**Authors:** Chenglin Lou, Alexandra M. Cross, Lien Peters, Daniel Ansari, Marc F. Joanisse

**Affiliations:** Department of Special Education, Peabody College of Education, Vanderbilt University, Nashville, TN, USA; Department of Psychology, The University of Western Ontario, London, Canada; Centre for Brain and Mind, The University of Western Ontario, London, Canada; Health and Rehabilitation Sciences, The University of Western Ontario, London, Canada; Faculty of Psychology and Educational Science, Department of Experimental Clinical and Health Psychology, Research in Developmental Disorder Lab, Ghent University, Ghent, Belgium; Faculty of Education, The University of Western Ontario, London, Canada; Haskins Laboratories, New Haven CT, USA

**Keywords:** Reading disabilities, Thalamus, Connectome, DTI, Topological properties, Reading network

## Abstract

We examined how thalamocortical connectivity structure reflects children’s reading performance. Diffusion-weighted MRI at 3 T and a series of reading measures were collected from 64 children (33 girls) ages 8–14 years with and without dyslexia. The topological properties of the left and right thalamus were computed based on the whole-brain white matter network and a hub-attached reading network, and were correlated with scores on several tests of children’s reading and reading-related abilities. Significant correlations between topological metrics of the left thalamus and reading scores were observed only in the hub-attached reading network. Local efficiency was negatively correlated with rapid automatized naming. Transmission cost was positively correlated with phonemic decoding, and this correlation was independent of network efficiency scores; follow-up analyses further demonstrated that this effect was specific to the pulvinar and mediodorsal nuclei of the left thalamus. We validated these results using an independent dataset and demonstrated that that the relationship between thalamic connectivity and phonemic decoding was specifically robust. Overall, the results highlight the role of the left thalamus and thalamocortical network in understanding the neurocognitive bases of skilled reading and dyslexia in children.

## Author Summary

The thalamus is increasingly considered a key component of the brain’s reading network, although its precise role is less well understood. Here, we apply a network neuroscience approach to understanding how the thalamus is embedded into the reading network by examining how its wiring pattern is associated with different component aspects of reading skills in children. Using a connectome framework, we show that a local efficiency metric of the left thalamus is negatively correlated with rapid automatized naming, while its transmission cost measure is positively correlated with phonemic decoding. Connections between temporal areas of cortex and two thalamic nuclei, the pulvinar and mediodorsal thalamic nucleus, were also negatively correlated with phonemic decoding. Our result thus provides clearer specificity about how the thalamus supports reading by coordinating communications across reading network areas.

## INTRODUCTION

Around 10% of children have developmental dyslexia or other reading disabilities (RD), with dysfluent and inaccurate reading performance that cannot be explained by deficits in intelligence (Lyon, Shaywitz, & Shaywitz, 2003). RD has been suggested as a disconnection syndrome associated with weaker functional connectivity between reading-related brain regions (Boets et al., 2013; Horwitz, Rumsey, & Donohue, 1998; Paulesu et al., 1996; Pugh et al., 2000). Likewise, previous studies using diffusion tensor imaging (DTI) have reported altered connectivity of white matter pathways within the reading network at the cortical level in RD, compared with typical readers (Cheema et al., 2023; Deutsch et al., 2005; Hoeft et al., 2011; Klingberg et al., 2000; Niogi & McCandliss, 2006; Odegard, Farris, Ring, McColl, & Black, 2009; Richards et al., 2008; Rimrodt, Peterson, Denckla, Kaufmann, & Cutting, 2010; Sihvonen, Virtala, Thiede, Laasonen, & Kujala, 2021; Steinbrink et al., 2008; Su et al., 2018; Vandermosten et al., 2012; Zhao, de Schotten, Altarelli, Dubois, & Ramus, 2016). However, there is also a growing interest in exploring the role of white matter connectivity involving subcortical regions. For example, the thalamus is the core of most sensory transmissions in the human neocortex. It has been suggested that the corticofugal connections that provide feedback from higher level to lower level area are essential to visual (Bullier, Hupé, James, & Girard, 2001; Juan, Campana, & Walsh, 2004; Ro, Breitmeyer, Burton, Singhal, & Lane, 2003) and auditory (Banai et al., 2009; Winer, 2005) sensory processing, and this feedback projection could also induce property changes of the thalamic neurons (Cudeiro & Sillito, 2006; Suga & Ma, 2003). As reading involves processing of visual and auditory information, the properties of the thalamus and thalamocortical connections may be altered in RD.

Associations between reading skills and functional activation in the left thalamus in the pulvinar have been demonstrated in fMRI studies of typical readers (Koyama, Molfese, Milham, Mencl, & Pugh, 2020; Pugh et al., 2013). In addition, recent fMRI studies in RD have reported altered functional activation in the left thalamus (Díaz, Hintz, Kiebel, & von Kriegstein, 2012; Paz-Alonso et al., 2018). These results are in line with observations from postmortem (Galaburda, Menard, & Rosen, 1994) and in vivo structural MRI studies (Giraldo-Chica, Hegarty, & Schneider, 2015; Jednorog et al., 2015), which have identified altered thalamic morphology in the left hemisphere in RD. It has been hypothesized that the observed alterations are related to abnormal white matter connections between the left thalamus and cortical regions in RD (Galaburda et al., 1994). Similarly, recent DTI studies have revealed alterations of white matter thalamocortical connectivity in RD (Davis et al., 2010; Fan, Davis, Anderson, & Cutting, 2014; Müller-Axt, Anwander, & von Kriegstein, 2017; Sihvonen et al., 2021; Tschentscher, Ruisinger, Blank, Díaz, & Von Kriegstein, 2019; Žarić et al., 2018), also indicated the involvement of the left thalamus. These include connectivity of the thalamus to a number of cortical areas including the superior temporal gyrus (STG), V5/MT, and inferior frontal gyrus (IFG).

The directions of group differences between typical readers and RD, and their associations with reading subskills, have also varied across studies. For example, studies in adults reported disrupted thalamocortical connections in RD compared with healthy controls and negative correlations between the connectivity of thalamocortical pathways and rapid naming times (Müller-Axt et al., 2017; Tschentscher et al., 2019). Studies in children, on the other hand, have identified higher thalamocortical connectivity and negative correlations between connectivity and reading performance (Davis et al., 2010; Fan et al., 2014; Žarić et al., 2018). Thus, it remains unclear what role the thalamus and thalamocortical connections play in reading and RD, and whether these findings are robust and replicable.

Here, we propose a connectome-based approach, which takes into account multiple thalamocortical regions and connections, to explore how the thalamus is involved in reading and RD. The connectome refers to the whole-brain connectivity network, with nodes and edges symbolizing cortical regions and connecting white matter tracts, respectively (Sporns, Tononi, & Kötter, 2005). This network is only sparsely connected, such that it relies on a subset of regions to act as interconnected hubs, which form a rich-club organization that facilitates long-distance signal transmission between cognitive modules (van den Heuvel & Sporns, 2011). Indeed, the thalamus is one of these hub regions (van den Heuvel, Kahn, Goñi, & Sporns, 2012; van den Heuvel & Sporns, 2011) and also coincides with the subset of interconnected regions forming the brain’s reading network (Lou, Cross, Peters, Ansari, & Joanisse, 2021).

As reading involves merging visual, phonological, and semantic information, the role of the thalamus as a hub might exert an important influence on reading skills. For instance, Bathelt, Gathercole, Butterfield, the CALM team, and Astle (2018) reported associations between the topological properties of hubs and reading achievement in school-age children with a wide range of reading abilities, suggesting the importance of maintaining a hierarchically organized network to support specific reading processes. Similarly, investigating particular connection patterns between hubs and reading network nodes in children with and without RD, we previously found that reading abilities are associated with connections between hubs and peripheral nodes in the left hemisphere (Lou et al., 2021). In the present work, we investigate more closely how the thalamus is embedded in the connectome and its contribution to specific reading skills.

At the network level, the properties of the network topology, such as efficient architecture and central communication hubs, can be quantitatively characterized by various graph theoretical parameters (Bullmore & Sporns, 2009). At the individual nodal level, graph theory can quantify how each node is embedded into either a local cluster of neighboring nodes or the global brain connectome, both of which indicate the importance of the node in information transmission. Regarding the local wiring pattern, the clustering coefficient (CC) is a basic measure that is equal to the fraction of closed triangles around the node (Watts & Strogatz, 1998), and local efficiency (LE) is determined by the shortest path length between each pair of neighboring nodes and generally evaluates the damage tolerance of the local cluster and communication efficiency among a node’s neighbors (Latora & Marchiori, 2001). The shortest path length demonstrates the cost of optimal routing strategy between any pair of nodes within the connectome. This routing model considers the global organization of the connectome, which is acknowledged by every node (Boccaletti, Latora, Moreno, Chavez, & Hwang, 2006; Goñi et al., 2013), but overlooks the contribution of local connections that constrain the spread of signal as a diffusion process (Masuda, Porter, & Lambiotte, 2017; Noh & Rieger, 2004). Recently, a stochastic routing model has been proposed describing routing as a continuous spectrum of changing weights between the shortest path and diffusion communication processes (Avena-Koenigsberger et al., 2019). It allows the evaluation of the communication cost from the spectrum of routing strategies as a dynamic measure compared with the shortest path length. Associations between those topological properties and reading abilities have been demonstrated in previous studies, which investigated whole-brain networks in various imaging modalities (Bathelt et al., 2018; Lou et al., 2019, 2021; Mao, Liu, Perkins, & Cao, 2021; Qi et al., 2016). However, whether these relationships could be observed on subcortical structures such as the thalamus, and how these relate to reading ability and disability, remains unknown.

Specifically, the thalamus is only rarely identified as a functional region when considering the network of cortical regions supporting reading (Bolger et al., 2005; Martin, Schurz, Kronbichler, & Richlan, 2015; Turkeltaub et al., 2002), even though some studies have identified differences in thalamic activity in good versus poor readers (e.g., Maisog, Einbinder, Flowers, Turkeltaub, & Eden, 2008; Pugh et al., 2013). Moreover, some recent studies have suggested a role of thalamocortical connectivity in explaining individual differences in reading (Achal, Hoeft, & Bray, 2016; Cross et al., 2021; Koyama et al., 2020). That said, most of these studies focus on how the thalamus connects to individual reading-related areas in the neocortex, which might provide an incomplete perspective given that reading engages a distributed network of regions. Here, we take a broader approach of measuring the topological properties of the left thalamus, including local wiring patterns and the cost of communication within a spectrum of routing strategies, in the connectome architecture. Considering the role of the thalamus as a hub region for between-module communication across spatially disparate brain regions allows us to explore if the thalamus is indirectly associated with reading by coordinating communication across the reading network.

We quantified this in each individual child and subsequently correlated it with their specific reading skills. Furthermore, since reading is a multicomponential skill, it is reasonable to expect that not all aspects of reading might equally rely on support from the thalamus. To address this, we specifically recruited a sample of children with a wide range of reading abilities. Moreover, we assessed specific component skills of reading such as phonological decoding, familiar word recognition, and reading comprehension or naming automaticity. As in previous studies, behavioral measures of reading were examined as continuous variables to capture the full spectrum of abilities, overcoming the ambiguous categorical distinctions between typical readers and individuals with RD/dyslexia. To validate the results, the same analyses were also applied to a second verification sample of early readers that captured both structural and diffusion MRI data and reading subskill measures in an independent sample of children (Lytle, McNorgan, & Booth, 2019).

## METHODS

### Participants

The primary sample included 73 native English-speaking children, aged between 8.83 and 14.68 years. There were 34 boys and 39 girls. According to the parental report, participants showed normal hearing and normal uncorrected vision abilities and had no history of neurological disorders. The children showed a wide range of reading abilities (Table 1). Eighteen participants had previously been identified as having reading difficulties by school professionals. Our sample showed a bias toward a greater number of poor readers in the younger age range. To address this, we removed nine older participants to eliminate confound between age and reading achievement. The final sample of 64 children showed no correlations between age and reading scores. This study was approved by the Western University Research Ethics Board. Informed consent was provided by a parent or guardian, and written or verbal assent was obtained from each child participant.

**Table 1.** Demographic and behavioral measures

	*Primary dataset*		*Validation dataset*	
	Mean/*N*	*SD*	Mean	*SD*
N	64		96	
Age (years)	10.94	1.26	10.92	1.58
Sex (boys/girls)	31/33		52/44	
Handedness (left/right)	2/62		0/96	
SWE (standard score)	93.02	21.35	98.72	14.21
Phonemic decoding (standard score)	92.72	19.83	97.01	15.28
Passage comprehension (standard score)	90.03	13.30	96.98	10.99
RAN (#correct/second)	1.87	0.48	1.99	0.50

A validation sample was also obtained from an open-access dataset of child readers (Lytle et al., 2019). This consisted of a total of 112 children, consisting of all children whose dataset included DTI scans. The demographic data and reading scores from this sample are listed in Table 1. Participants with excessive motion (more than 10 volumes showing a displacement of 3 mm or 3° of movement in any direction) were excluded from the data, leading to 96 children included in the present study.

### Behavioral Measures

Participants in both samples completed a series of standardized tests measuring reading subskills. The Test of Word Reading Efficiency 2 (TOWRE; Torgesen, Wagner, & Rashotte, 2012) Sight Word Efficiency (SWE) subtest examined single-word reading fluency. The TOWRE Phonemic Decoding Efficiency (PDE) subtest was used to measure decoding fluency. The Passage Comprehension subtest of the Woodcock-Johnson III (Woodcock, McGrew, & Mather, 2001) was used to measure reading comprehension. Rapid naming was assessed differently in the two samples; the primary sample performed rapid automatized naming (RAN) of letters, in which they viewed the letters K, R, M, and G presented repeatedly in a random order in a 5 × 10 grid and named each item as quickly as possible (Arnell, Joanisse, Klein, Busseri, & Tannock, 2009). Children were scored on correctly named items per second. The validation group performed the Digit and Letter components of the Rapid Symbolic Naming subtest of the Comprehensive Test of Phonological Processing (Bruno & Walker, 1999). Note that because the items and scoring method of these two measures of RAN are slightly different, some caution was taken when comparing the two samples on this test, as discussed in the Results section.

### Imaging Acquisition

#### Primary sample.

To familiarize children with the scanner environment and assess whether each child could remain sufficiently still during MRI scanning, a training session was conducted for each child before MRI scanning. Participants were asked to lie still on a bed inside a simulated MRI scanner and listened to an audiobook while the MRI scanner noise was played via loudspeakers for around 5 min. Feedback was provided to participants about head movement using an electromagnetic motion tracker. No participants were excluded due to excessive movement or reported discomfort during the training session. Participants then moved on to the actual MRI scanning with a Siemens 3 T Magnetom Prisma MRI scanner equipped with a 32-channel head coil at the Robarts Research Institute, Western University, London, Ontario, Canada. Foam pads were used during the scanning to control head movement. A T1-weighted MPRAGE sequence (TR = 2.30 s, TE = 2.98 ms, FOV = 256 mm × 256 mm, voxel size = 1 mm × 1 mm × 1 mm, 192 slices) was used for whole-brain three-dimensional anatomical imaging. The DTI sequence consisted of diffusion-weighted (DW) images (TR = 3 s, TE = 50.6 ms, FOV = 256 mm × 256 mm, voxel size = 2.04 mm × 2.04 mm × 2 mm, 64 slices, 56 directions with b = 1,000 s/mm^2^ and 8 directions with b = 0). Additional fMRI scans were also acquired within the same session, but not reported here.

#### Validation data.

Participants in the validation dataset also performed an MRI training session prior to actual imaging. During actual MRI scanning, foam pads were used to minimize head movement. Images were acquired using a 3 T Siemens Tim Trio scanner equipped with a 16-channel head coil at Northwestern University Center for Advanced Magnetic Resonance Imaging. A T1-weighted Magnetization Prepared - RApid Gradient Echo (MPRAGE) sequence (TR = 2.30 s, TE = 3.36 ms, FOV = 256 mm × 256 mm, voxel size = 1 mm × 1 mm × 1 mm, 160 slices) and DTI sequence (TR = 9.4, 9.5 or 9.512 s, TE = 89 ms, FOV = 256 mm × 256 mm, voxel size = 2 mm, 72 slices, 64 directions with b = 1,000 s/mm^2^ and 1 direction with b = 0) were used to acquire whole-brain anatomical and DW images, respectively.

### Image Processing

The p-code version of the ExploreDTI (Leemans, Jeurissen, Sijbers, & Jones, 2009; https://www.exploredti.com) toolbox for MATLAB was used for processing DW images. Head motion and eddy current correction were applied to raw DW images. These head motion parameters were also used to examine head movement, confirming that no participants or images needed to be excluded due to excessive head motion. Images were then nonlinearly registered onto the T1 images to correct EPI distortions.

The nodes of the connectome were delineated based on T1-weighted images as follows. T1-weighted images were skull-stripped using the FSL Brain Extraction Tool (Smith, 2002), and the remaining brain images were parcellated into 90 gray matter regions of interest (ROI) based on the Automated Anatomical Labeling template (Tzourio-Mazoyer et al., 2002). It was completed by registering the template onto the skull-stripped brain image of each participant using FMRIB’s Nonlinear Image Registration Tool (FNIRT, FSL; Jenkinson, Beckmann, Behrens, Woolrich, & Smith, 2012; https://www.fmrib.ox.ac.uk/fsl/) via the following steps. The FA map of each participant from the corrected DTI image was registered to the skull-stripped images to acquire a transfer matrix. Next, the skull-stripped images were nonlinearly registered to the MNI (Montreal Neurological Institute) 152 template. The two transfer matrices were combined and inverted to generate a transformation matrix from the standard space to each participant’s native space. The automated anatomical labeling (AAL) template in each native space was then acquired by applying the matrix to the template.

As for the edges (connections) of the connectome, whole-brain tractography was performed to reconstruct white matter fibers based on corrected DTI images. A deterministic DTI algorithm was applied to perform the fiber tracking with the following rules: White matter fibers were tracked from each voxel as a seed in each node to all other nodes, following the main diffusion direction until (a) encountering a voxel with fractional anisotropy lower than 0.1, (b) the turning angle was higher than 60°, or (c) the range of fiber length was limited between 25 and 500 mm with a step size of 0.5 mm.

Our first-pass analyses followed the AAL atlas, which labels the thalamus in each hemisphere as a single region. However, the thalamus consists of multiple nuclei that connect to distinct cortical areas. To examine more closely how the connectivity of these distinct subregions of the thalamus contribute to reading ability, we parcellated the thalamus into 12 subregions in each hemisphere following the Thalamus Optimized Multi Atlas Segmentation (THOMAS). This approach uses an automated segmentation approach to identifying thalamic nuclei (Su et al., 2019). Connections between each subregion of the thalamus and cortical regions were then quantified based on the same deterministic tractography approach as above, with the same thresholding rule that removed connections whose number of streamlines were lower than 3. However, the finer partitioning could lead to difficulties in successfully tracking their connections toward cortical regions. Therefore, we also reconstructed connections between each thalamic subregion and the cortical areas of interest using probabilistic tractography, which has been applied in previous studies of subregions of the thalamus such as lateral geniculate nucleus (LGN; Müller-Axt et al., 2017), medial geniculate body (MGB; Tschentscher et al., 2019), and mediodorsal thalamic nucleus (MD; Li et al., 2022). We applied the default fiber tracking parameters (step size = 0.5 mm, maximum of 2,000 steps, maximum turning angle = 0.2 rads, 5,000 tracks generated from each seed voxel). Normalized index of connectivity was applied based on the number of voxels in the seed region:

$$I=\log\left(\text{waytotal}\right)/\log\left(5000*V_{\text{seed}}\right)$$

where *waytotal* indicates the total number of streamlines from seed to target regions after the permutation, and *V*_*seed*_ refers to the number of voxels in the seed region.

### Network Analysis

#### Topological properties of the thalamus.

To minimize false-positive tracts between connectome nodes and to yield a sparsely connected matrix, edges with the fewer than three streamlines were removed. Two aspects of topological properties of the thalamus, efficiency and routing cost, were estimated for each participant’s whole-brain network, as well as for a hub-attached reading network composed of hubs and reading network nodes. The definition of hubs and reading network nodes was based on a prior study (Lou et al., 2021): Using the connectome of each participant, a node was considered a hub if its degree coefficient (number of other directly connected nodes) was higher than 20. The list of reading network regions included pars triangularis and pars opercularis of IFG, insula, fusiform gyrus, supramarginal gyrus, angular gyrus, Heschl’s gyrus, STG, middle temporal gyrus (MTG), inferior temporal gyrus, superior temporal pole, Rolandic operculum, and the thalamus in the left hemisphere.

In the context of graph theory, efficiency describes how the information is efficiently exchanged within the network (Latora & Marchiori, 2001). For each node in the connectome, its efficiency evaluates how the information is transported within clusters around the node (Rubinov & Sporns, 2010). The present study estimated the efficiency of the left thalamus using the standard graph-theoretic measures of LE and CC. While those two measures are usually highly correlated, the properties of the connectome that they describe are slightly different. LE measures how efficiently information is exchanged over the subnetwork that is directly connected with the node. CC, as one of the measures of network segregation, quantifies the prevalence of closed triangles that includes the node as one vertex, suggesting the potential for functional segregation within the network (Rubinov & Sporns, 2010).

**Figure 1.**
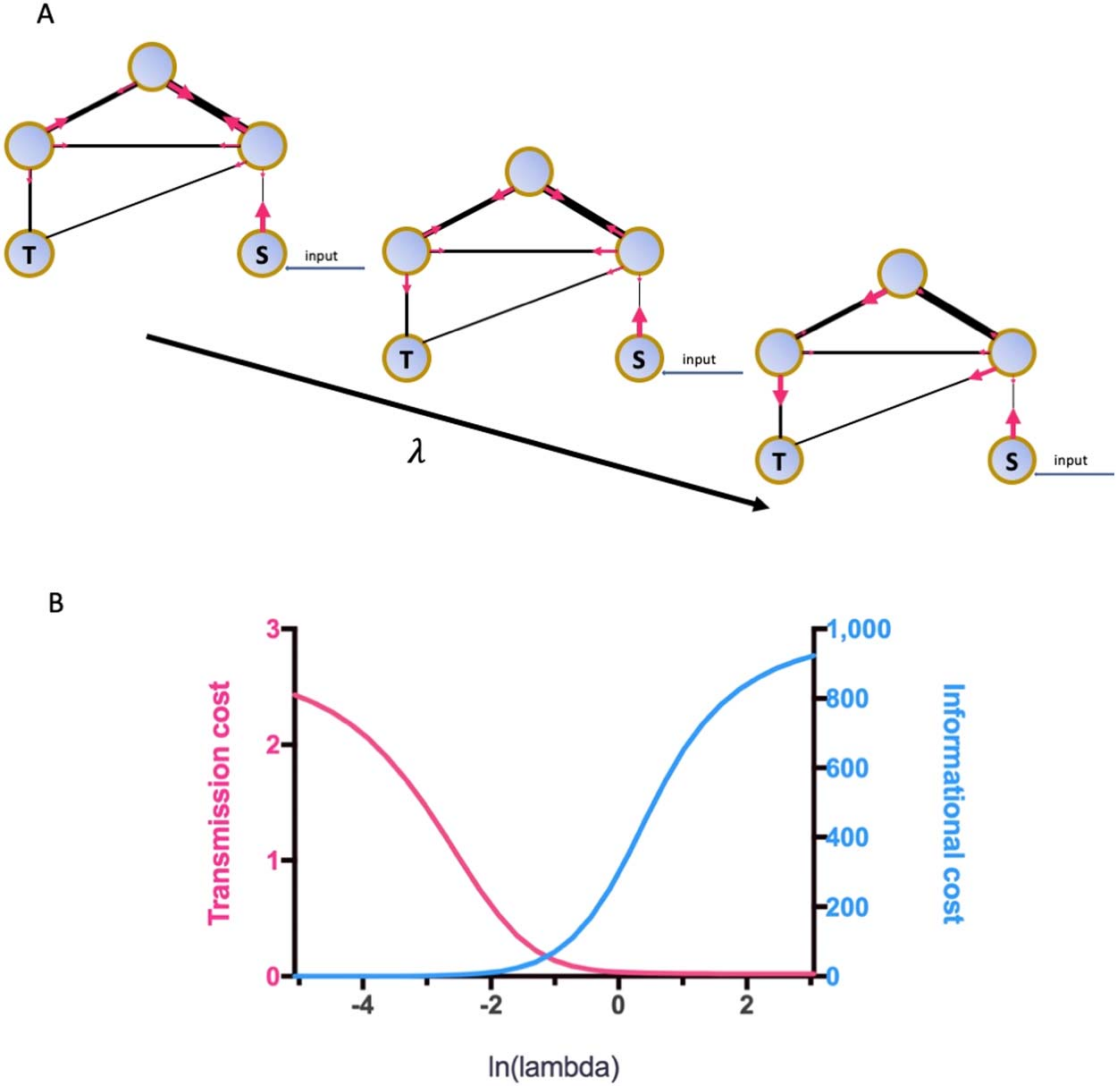
Dynamics of the routing strategy along with the changes of weights on global shortest path information. (A) Demonstration of the transition probabilities from one node to the next node within a spectrum of lambda values. Lambda increases along with the direction with the arrow. S refers to the node that receives the input and where the information transmission starts; T refers to the target node. The width of the black lines indicates the strength of the connection between the nodes it links. The pink arrow refers to the direction of the information flow, and the size of the arrow indicates the possibility of the transmission along with the direction of the arrow. The information flow prefers to move along with the strongest connections when the lambda is low, while the movement is more possible toward the target node directly when the lambda increases. (B) Averages of *C*^*trans*^ (pink) and *C*^*info*^ (blue) across all node pairs, as a function of lambda.

The routing strategy is depicted via a stochastic model for network communication that combines the local connectivity and global shortest path length, demonstrating biased random walks on the connectome (Avena-Koenigsberger et al., 2019). For each signal transferring from the current node **X** to a target node **T**, the routing strategy refers to a map containing every available route and the transition possibility at any node that the signal would move to the next node **Y**. As the routing strategy model combines both local and global topology of the network, transition probabilities at every node were defined as a dynamic system where the global topology was tuned by a parameter *λ*:

$$P_{\lambda }\left(Y=j|X=i,T=t\right)=\text{exp}\left(-\left(\lambda \left(d_{ij}+g_{jt}\right)+d_{ij}\right)\right)\frac{1}{Z_{i}^{t}}$$

where $Z_{i}^{t}$ = ∑_*j*_ *exp*(−(*λ*(*d*_*ij*_ + *g*_*jt*_) + *d*_*ij*_)) is a normalization factor. The local topology *d*_*ij*_ refers to the connectivity between node *i* and *j*, and the global topology *g*_*jt*_ refers to the shortest path between node *j* and node *t*. With the increase of the *λ*, the routing strategy gradually changes from an unbiased walk to a shortest path walk (Figure 1A). The present study applied the space of *λ* based on Avena-Koenigsberger et al. (2019), with a range from 1.45 to −2.2 in the logarithmic space and 30 equally spaced steps. Based on the transition probabilities, two communication costs of the routing strategy were characterized, transmission cost *C*^*trans*^ and informational cost *C*^*info*^. The transmission cost between the source node **X** and the target node **T** evaluates the cost related to signal transmission from one node to its neighbors, which is equivalent to the expected walking distance under the routing strategy. The informational cost evaluates the cost related to the usage of global shortest path information for efficient message transmission (Avena-Koenigsberger et al., 2019). It is quantified by the Kullback-Leibler divergence between the current routing strategy and the null model routing strategy, which measures the bits of information required for applying the routing strategy that is different from the null model. *C*^*trans*^ is decreasing along with the increasing lambda, while *C*^*info*^ is increasing, as the routing strategy is more weighted on the global shortest path information (Avena-Koenigsberger et al., 2019). As shown in Figure 1B, the dynamics of the two cost measures acquired from data in the present study followed the trend.

For each type of cost, the cost of traveling from the source to the target nodes could be extracted between any pair of nodes. It should be noted that for any pair of nodes, the cost may not be the same when the source node is switched with the target node. To evaluate the cost of transmission *C*_*thal*_ between any pair of reading network regions through the left thalamus, the left thalamus was set as a temporary target and source node:

$$C_{\text{thala}}=C_{RN\to \text{thal}}+C_{\text{thal}\to RN}$$

where *C*_*RN*→*thal*_ refers to the sum of cost with each reading network region as the source node and the left thalamus as the target node, and *C*_*thal*→*RN*_ refers to the sum of cost with the left thalamus as the source node and each reading network region as the target node. Both transmission cost $C_{\text{thal}}^{\text{trans}}$ and informational cost $C_{\text{thal}}^{\text{info}}$ of the left thalamus were then computed. To identify the range of lambda values where the communication cost of the empirical connectome was more efficient, normalized cost measures ‖*C*^*trans*^‖ = *C*^*trans*^(*emp*)/*C*^*trans*^(*rand*) and ‖*C*^*info*^‖ = *C*^*info*^(*emp*)/*C*^*info*^(*rand*) were calculated under each *λ*. It compared the cost measures of the observed connectome with the measures of its randomized counterparts that preserved the node degree, strength, and weight distribution of the network. The *λ* value where the cost measures were lower in empirical than randomized networks was highlighted for transmission and informational cost measures, respectively. $C_{\text{thal}}^{\text{trans}}$ and $C_{\text{thal}}^{\text{info}}$ were extracted, and we averaged the cost measures across the range of ranges of *λ* where the normalized cost values were lower than 1. Specifically, the transmission cost was averaged across the range of ln(*λ*) between −5.1 and 0.15, and the informational cost was averaged across the range of ln(*λ*) between −0.14 and 2.76.

Aside from the connectomic properties of the left thalamus, the present study also investigated the strength of thalamocortical connections in the left hemisphere, with the number of connections between the thalamus and reading network regions per edge being extracted. In addition, we also performed the congruent analyses with right hemisphere regions, in order to examine the specificity of the results to left hemisphere thalamus and cortical regions.

### Statistical Analysis

We examined correlations between efficiency indices of the left thalamus in the whole-brain, connectome hubs and in the reading network, and the behavioral measures of reading. As for the routing strategy, the two indices of cost were averaged across a range of *λ* where the corresponding normalized cost measures were lower than 1, which indicated that the routing strategy is more efficient than a random network within the range. Correlations between the mean cost of the two indices and reading scores were then examined. In all cases, partial correlations were used controlling for sex, mean FA of all edges within the hub-attached reading network, and handedness. As the RAN score was not normalized by age, partial correlations were calculated between RAN and network measures, with age as a covariate. Because of the large number of tests being conducted, family-wise error was corrected with 10,000-permutation Monte Carlo simulations.

To test if the communication cost could explain the variance of reading scores beyond the commonly investigated efficiency measures, a hierarchical multiple regression analysis was performed. The reading scores that were significantly correlated with communication cost measures were the dependent variable. Three models were applied while the following parameters were entered as independent variables. Model 1 included sex, the mean FA of all edges connecting the left thalamus and handedness, which were covariates in correlation analyses. Model 2 included Model 1 and the two efficiency measures of the left thalamus. Model 3 contained Model 2 and the communication cost measures of the left thalamus.

## RESULTS

### Connectome Properties

Before performing correlations between the connectome properties and reading scores, we examined whether age was confounded with connectome measures or normalized reading scores. Results showed that age was not correlated with any measures (∣*r*∣*s* < 0.24, *ps* > 0.06).

**Figure 2.**
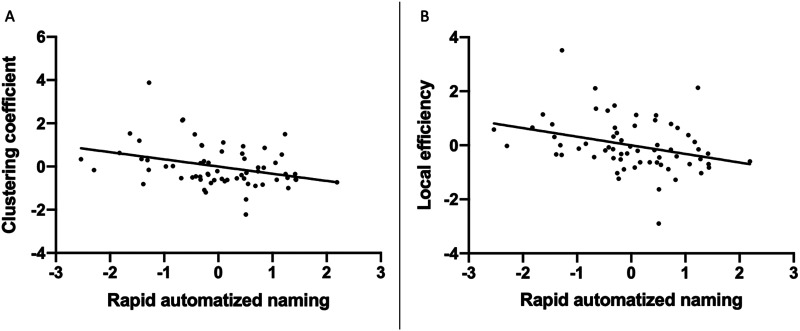
Correlations between RAN and (A) CC and (B) LE of the left thalamus. RAN was scored as correctly answered items per second. Plotted values are standard residuals after controlling for age, sex, mean FA within the hub-attached reading network including reading network and hub regions, and handedness.

For the efficiency measures, both CC (*r* = −0.29) and LE (*r* = −0.30) of the left thalamus in the hub-attached reading network were negatively correlated with RAN (Figure 2). However, these effects did not survive correction for multiple comparisons. Word reading (TOWRE PDE) and passage comprehension scores were also negatively correlated with any of the two efficiency measures in the hub-attached reading network, while none of them was survived the correction either. Congruent analyses examined reading subtest scores and efficiency measures of the left thalamus within the whole-brain network. These revealed no significant results.

**Figure 3.**
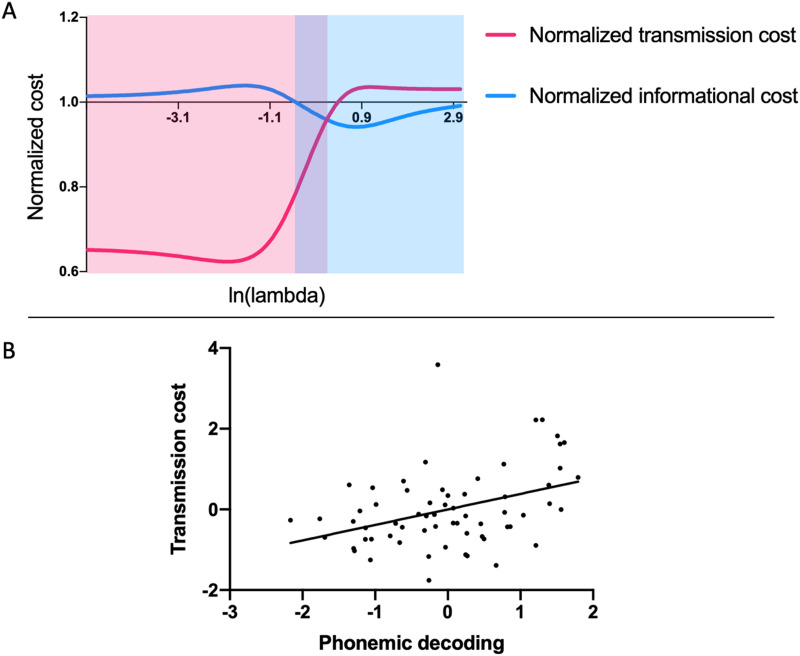
(A) Averages of ‖*C*^*trans*^‖ (pink) and ‖*C*^*info*^‖ (blue) across all node pairs. The curves are computed by normalizing *C*^*trans*^ and *C*^*info*^ concerning the same cost measures computed on ensembles of 500 randomized networks (per subject). Shaded pink and blue areas indicate sections of the ‖*C*^*trans*^‖ and ‖*C*^*info*^‖ curves that are smaller than 1, respectively, indicating the regions in the spectrum where the communication cost of empirical networks (i.e., networks that are derived from empirical data) is smaller than the cost computed on the randomized ensembles. The overlapped area (dark blue) indicates sections of the curves ‖*C*^*trans*^‖ and ‖*C*^*info*^‖ that are both smaller than 1. (B) Correlations between the PDE score and the transmission cost of the left thalamus, which was the mean transmission cost across areas where ‖*C*^*trans*^‖ was lower than 1. Plotted values are standard residuals after controlling for sex, mean FA within the hub-attached reading network including reading network and hub regions, and handedness.

Regarding the routing cost measures, the transmission cost of the left thalamus within the hub-attached reading network was positively correlated with the TOWRE PDE score (*r* = 0.34, corrected *p* = 0.041) (Figure 3B). No reading subskill tests were correlated with the transmission cost of the left thalamus across the whole-brain network. The informational cost of the thalamus, either within the hub-attached reading network or across the whole-brain network, was not correlated with any reading subskill scores.

### Between-Metric Correlations

As TOWRE PDE and transmission cost were the pair of measures showing a significant correlation, a hierarchical multiple regression was applied with PDE as the dependent variable and the transmission cost was put into Model 3. As shown in Table 2, the transmission cost was still significantly correlated with the PDE score after the variation, which could be explained by efficiency measures being excluded.

**Table 2.** Hierarchical multiple regression analyses, with the PDE score as the dependent variable

	*R* ^2^	Δ*R*^2^	*F*	*p*
Model 1: Sex, handedness, and mean FA of the thalamocortical connection in the hub-attached reading network	0.120	0.120	2.72	0.052
Model 2: CC and LE of the left thalamus	0.204	0.084	3.042	0.055
Model 3: Transmission cost of the left thalamus	**0.289**	**0.086**	**6.88**	0.011*

Significant model is **bolded** and labeled with *.

To examine whether the results were specific to the left hemisphere, the hub-attached reading network regions in the right hemisphere were selected and the same statistical analyses were applied. However, no significant results were found between topological measures and reading subskill scores.

### Subregions of the Thalamus

As significant correlations were only observed in the hub-attached reading network, we focused on connections between the subregions of thalamus and reading network areas to explore divergence of their contributions to reading. First, Table 3 shows the success rates of tracking between each subregion of the thalamus and any of reading network regions using deterministic tractography. Among all 24 subregions of the bilateral thalamus, a few connections to the right thalamus were reconstructed. For the left thalamus, tracking toward the pulvinar and MD showed a higher success rate and reliability than the rest of the subregions of the thalamus. In addition, these two regions connect different regions within the reading network. Therefore, we chose the pulvinar and MD as the ROI for follow-up measures of probabilistic tractography connections.

**Table 3.** Success rate of tracking connections using deterministic tractography between subregions of the thalamus and reading network regions across 64 participants

	Left hemisphere	Right hemisphere
Anterior ventral nucleus	17.4%	0%
Ventral anterior nucleus	3.2%	0%
Ventral lateral anterior nucleus	0%	0%
Ventral lateral posterior nucleus	11.1%	0%
Ventral posterior lateral nucleus	0%	0%
Pulvinar nucleus	**88.9%**	9.5%
Lateral geniculate nucleus	14.3%	0%
Medial geniculate nucleus	20.6%	4.8%
Centromedian nucleus	6.3%	0%
Mediodorsal nucleus	**58.7%**	1.6%
Habenular nucleus	0%	0%
Mammillothalamic tract	7.9%	1.6%

Connections with number of streamlines lower than 3 were removed.

Mean connectivity of normalized probabilistic connections from both pulvinar and MD to all reading network areas was negatively correlated with phonemic decoding (TOWRE PDE; *r* = −0.31, *p* = 0.017 and *r* = −0.26, *p* = 0.041, for pulvinar and MD, respectively), using a partial correlation controlling for age. A congruent analysis showed no effect for the RAN score. To examine which specific thalamo-reading-network connections drive the significant effects, partial correlation tests were conducted between the TWORE PDE score and normalized probabilistic connections between the pulvinar/MD and each reading network regions. For both pulvinar and MD, their connections to temporal areas, including STG (pulvinar: *r* = −0.38, *p* = 0.003; MD: *r* = −0.35, *p* = 0.006), MTG (pulvinar: *r* = −0.27, *p* = 0.036; MD: *r* = −0.29, *p* = 0.022), and superior temporal pole (pulvinar: *r* = −0.28, *p* = 0.031; MD: *r* = −0.31, *p* = 0.016), were all significantly correlated with the PDE score. Connections to other reading network areas were not correlated with the PDE score.

### Validation Sample Results

**Figure 4.**
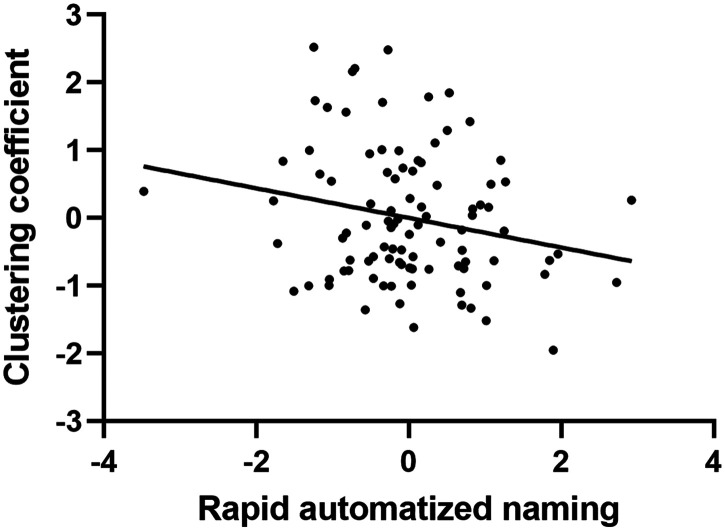
Correlations between RAN and CC of the left thalamus in the validation. The RAN was scored as correctly answered items per second. Plotted values are standard residuals after controlling for age, sex, and mean FA within the hub-attached reading network including reading network and hub regions.

Results from the open-access dataset replicated several significant findings on the topological properties of the left thalamus. Specifically, while the negative correlations between the two network measures and the RAN letter naming raw score did not survive the multiple comparison corrections, the validation data showed that the number of named letters per second was significantly correlated with CC (*r* = −0.219, *p* = 0.035; Figure 4) but not with LE (*r* = −0.185, *p* = 0.076). No other significant correlations were observed in the validation. In terms of the routing strategy, the transmission cost of the left thalamus was positively correlated with the PDE score (*r* = 0.239, *p* = 0.020). Similarly, a hierarchical regression showed results congruent with those in the primary analysis (Table 4). No significant correlations were observed in topological measures within the whole-brain network. Regarding analyses of subregions of the thalamus, the success rate of tracking connections between MD and the reading network was 32.4% after thresholding. The success rate of tracking connections between the pulvinar and reading network was still robust, at 92.5% after thresholding. No significant correlations were found in the validation dataset. As discussed further below, this may reflect important differences in MRI hardware employed in acquiring the primary versus secondary datasets.

**Table 4.** Hierarchical multiple regression analyses, with the PDE score as the dependent variable

	*R* ^2^	Δ*R*^2^	*F*	*p*
Model 1*: Aex and mean FA of the thalamocortical connection in the hub-attached reading network	0.041	0.041	1.98	0.144
Model 2: CC and LE of the left thalamus	0.081	0.040	2.00	0.101
Model 3: Transmission cost of the left thalamus	**0.126**	**0.045**	**4.66**	**0.031** *

Handedness was not included because all participants were right-handed in the validation dataset.

Significant model is **bolded** and labeled with *.

## DISCUSSION

This study examined the topological properties of the thalamus in the left hemisphere and their relationship with reading performance in children with and without RD. Reading involves mapping orthography, phonology, and semantics via separate lexical and sublexical pathways (Coltheart, Curtis, Atkins, & Haller, 2013; Jobard, Crivello, & Tzourio-Mazoyer, 2003; Seidenberg & McClelland, 1989). While studies investigating the neural correlates of those pathways usually focus on cortical areas, the thalamus has also been demonstrated contributing to reading functions. The thalamus serves as an information relay station that is involved in multiple cognitive functions (Hwang, Bertolero, Liu, & D’Esposito, 2017). In reading, it has been suggested that the thalamus is involved in processing and transmitting sensory input toward cortical regions, and the deficit of the thalamocortical connections might be the origin of RD (Galaburda, 1993; Galaburda et al., 1994; Stein & Walsh, 1997). Altered connections between the thalamus and reading-related areas have been reported in dyslexia/RD both in fMRI (Koyama et al., 2020; Paz-Alonso et al., 2018) and DTI studies (Fan et al., 2014; Müller-Axt et al., 2017; Tschentscher et al., 2019; Žarić et al., 2018). In addition, thalamocortical connections have been associated with component measures of skilled reading, including RAN, phonological decoding, and letter-word identification (Davis et al., 2010; Fan et al., 2014; Müller-Axt et al., 2017; Pugh et al., 2013; Tschentscher et al., 2019; Žarić et al., 2018). Those studies provide a map of the relationship between the way the thalamus interacts with cortical nodes and variations in reading abilities.

The current study evaluated more closely the wiring patterns of the left thalamus and reading network regions using graph-theoretical approaches and linked these associations with specific reading subskills. Negative correlations were found between rapid naming (RAN) and CC, which describe how the thalamus was connected with its surrounding nodes. While the correlation between RAN and CC did not survive correction for multiple comparisons in the primary dataset, it was confirmed in the validation dataset that employed a similar RAN measure. We found a more robust effect of nonword decoding and thalamic connectivity: The transmission cost of the left thalamus within the hub-attached reading network was positively correlated with the standardized nonword decoding (measured with TOWRE PDE), even after the variation of these scores that could be explained by the two efficiency measures was removed. All three of these findings were also replicated with a secondary dataset acquired from an independent sample of young readers within a similar age range, supporting the view that this finding was not idiosyncratic of the children in our dataset.

**Figure 5.**
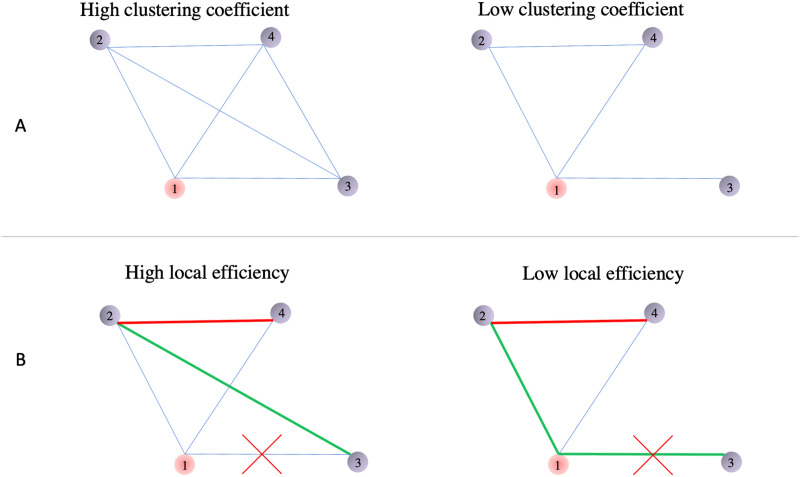
Demonstration of the changes of CC and LE. (A) The CC of one node (red) is measured according to the prevalence of closed triangles among the central node and its immediate neighbors (gray). The higher CC of node 1 encloses three closed triangles (1-2-3, 1-2-4, 1-3-4; left panel) out of three possible triangles, while the lower CC of node 1 encloses one closed triangle (1-2-4; right panel) out of three possible triangles. (B) The LE of one node (red) measures the fault tolerance of the subnetwork. The green and red lines represent the shortest path between nodes 2 and 3 as well as nodes 2 and 4, respectively. When the disconnection occurs on path 1-3, the communication between nodes 2 and 3 is less affected with higher LE (left panel), whereas it is completely disrupted with a lower LE (right panel).

Importantly, the results suggest that these variations in thalamocortical connections can be observed at the connectome level, which merges multiple connections into one single system, and that reading subskills could be explained by different topological properties of the system. CC, the nodal graph theoretical measure, sketches the accessibility from any node to another in different approaches. Negative correlations between the topological measure and RAN score suggest that children with better performance in RAN exhibited lower efficiency for information exchange within the thalamus-centered cluster. Previous studies mostly identified positive correlations between reading/language skills and efficiency measures at both global and nodal levels (Bathelt et al., 2018; Lee, O’Hara, Behen, & Jeong, 2020; Lou et al., 2019). The counterintuitive findings in the present study pinpoint a less-completed connection environment surrounding the left thalamus in better readers. Meanwhile, the importance of the thalamocortical connections for reading abilities is simultaneously highlighted. Specifically, lower CC means fewer closed triangles within a cluster that is centered with the primary node (Figure 5A). This indicates that the thalamus-centered cluster with a lower CC requires the information flow to pass through the thalamus more frequently, and the disconnection of the thalamocortical connections would have more significant consequences. Therefore, the negative correlation suggested that children who scored higher on RAN rely more heavily on direct neural connections between the thalamus and cortical reading network regions. In addition, LE is more sensitive to variations of the shortest path (Figure 5B), which is not the only routing strategy for information transmission. Therefore, it may be the reason why the correlations between LE and RAN score was not significant in either primary or validation dataset.

In addition, the efficiency measures of individual node assess the presence of a functionally segregated cluster, which is formed for specialized cognitive processing (Rubinov & Sporns, 2010). The thalamus is one of a relatively small number of hub nodes and is associated with multiple cognitive functions (Hwang et al., 2017). The connection pattern of the system follows a principle of conservation of physical wiring costs (Bullmore & Sporns, 2009; Chklovskii, Schikorski, & Stevens, 2002; Klyachko & Stevens, 2003), and the brain hubs usually exhibit low clustering in the connectome (Sporns et al., 2005; van den Heuvel et al., 2012). Higher CC in readers with a lower RAN score indicated that additional physical support beyond a concise thalamus-centered network was required in poor readers, reflecting a violation of the wiring principle. The negative correlation was identified only in the hub-attached reading network, underlining that the effect could be observed within the specific local subsystem. In addition, only children’s RAN scores were correlated with the two efficiency measures. The RAN test is unique in examining the ability to rapidly identify and name single letters or other objects, independent of higher level processing such as decoding and semantic processing typical of word-level tasks (Norton & Wolf, 2012), which might be more dependent on corticocortical connections. It also involves multisensory integration of visual letters with their corresponding pronunciations, which is associated with direct connections between the thalamus and sensory cortices (Hwang et al., 2017). As the efficiency measures evaluated clusters, which were composed of the thalamus and its immediate neighboring nodes, they can capture variations of the reading skills that were dependent more on the thalamocortical connections. Furthermore, RAN also requires sustained attention over time and coordinating eye movements with articulations (Wolf & Bowers, 1999), which require thalamic connections (Crick, 1984; Rafal & Posner, 1987; Wimmer et al., 2015).

Pseudoword reading, here measured using the TOWRE PDE task, is crucial for making connections between novel letter sequences and an acquired phonological lexicon. The positive correlation between the PDE score and transmission cost indicated higher involvement or coordination of these multiple brain regions during this task in better readers, as the transmission cost is mathematically equal to the expected distance of all possible routes from source to target nodes (Avena-Koenigsberger et al., 2019). A meta-analysis, which summarized 53 studies in dyslexia using positron emission tomography (PET) or fMRI, reported decreased activation during reading and reading-like tasks within the reading network regions in dyslexics, indicating the insufficient commitment to reading within the reading network areas in dyslexics (Paulesu, Danelli, & Berlingeri, 2014). The present study supports these findings at the structural connectome level, suggesting that the lack of reading network engagement in RD is accompanied by altered white matter connections centered by a reading-related hub—the thalamus. The reading input of pseudowords needs to be comprehensively processed before being transmitted to the hub and after being dispersed to different reading modules.

Unlike SWE, PDE involves only sublexical processing, which is an attention-demanding process (Reynolds & Besner, 2006). It needs to divide a letter string into separated graphemes, requiring an orienting of visual spatial attention (Cestnick & Coltheart, 1999; Facoetti et al., 2006; Perry, Ziegler, & Zorzi, 2007). The nonsignificant correlation between transmission cost and the SWE score may accordingly originate from less engagement of visual attention, as the thalamus also plays a role in directing attention in visual space (Crick, 1984; Rafal & Posner, 1987; Wimmer et al., 2015). In addition, it may be due to words in SWE being more familiar than pseudowords in PDE. The processing of short-term sensory input in the thalamus is modulated by the predictability of the stimulus (Díaz, Hintz, Kiebel, & von Kriegstein, 2012). Better readers could predict more accurately, which benefits the performance (Denckla & Rudel, 1976; Landerl, Wimmer, & Frith, 1997; Tallal, 1980), leading to preserved cost in this situation. Therefore, it might be another origin that compromised the positive cost-reading correlation in other reading tests.

Transmission cost is also considered an indirect measure of the metabolic cost of transmitting neural signals from the source to the target nodes, as determined by the length of the communication pathways (Avena-Koenigsberger et al., 2019). A higher transmission cost between reading network regions that pass through the thalamus suggests a higher metabolic energy, as the metabolic cost of the brain is attributable to the maintenance of electrochemical gradients across neuronal membranes, which supports signaling and coordination of neuronal activity (Attwell & Laughlin, 2001; Niven & Laughlin, 2008). Accordingly, previous findings that demonstrated higher functional activation in the thalamus in readers with better nonword decoding scores (Pugh et al., 2013) could also be an indicator of a structurally determined metabolic cost.

In a follow-up analysis, we segmented the thalamus into multiple finer partitions to better capture how different thalamic nuclei connect to different cortical subregions. Accordingly, we found connections between temporal areas in the reading network and the pulvinar and MD to both be significantly associated with the TOWRE PDE measure of measure of phonemic decoding. Negative correlations indicated that children with poor decoding skills had more connections between the pulvinar/MD and temporal regions, which are responsible for phonological processing. This may due to the fact that the thalamus mediates interactions between reading-related regions rather than being directly responsible to specific reading processes. For example, decreased fMRI activation in the pulvinar during phonological tasks has been identified in children with RD (Gaab, Gabrieli, Deutsch, Tallal, & Temple, 2007; Pugh et al., 2013). In addition, Pugh et al. (2013) also observed the involvement of visual areas during phonological processing of words. They suggested that the extensive connections between pulvinar and the visual network support orthographic learning, which is also constrained by phonological, morphological, and semantic knowledge. Therefore, the authors proposed that the pulvinar mediates selective attention to the various sources of features, which are propagated from ventral regions like the fusiform and temporal areas that support orthographic learning. Stronger connections between the thalamus and temporal regions in poor PDE children may reflect their deficits in phonological processing and the need for more connections to support phonological processing during reading. Similarly, this could be applied to the MD subregion of thalamus, which has extensive connections to the prefrontal subregions of the reading network including pars triangularis and pars opercularis.

We do note that, unlike our other results, this analysis of thalamic subregions did not replicate in a secondary validation dataset. This appears to be related to more general difficulties in fine-grained tracking of fibers that connect deep structures such as the thalamus. For example, the success rate for tracking connections between MD and reading network regions was 32.43% in secondary validation dataset compared with 77.48% in our primary dataset. We suggest that this relates to subtle but important differences in the MRI hardware used to acquire these datasets. While both were acquired at 3 T, the primary dataset was acquired using an upgraded gradient, head coil and shimming systems compared with what was used in the verification dataset. In particular, the use of a 32-channel head coil in the primary dataset as well as shorter repetition and echo times (TR/TE) may have improved signal-to-noise ratio in the primary versus secondary datasets. The results suggest that more advanced DW image acquisition sequences, diffusion computation models, and tractography algorithms may be keys for reconstructing robust connections to deep cortical structures and for fine-grained parcellations of thalamic nuclei.

### Conclusion

The present study examined how the left thalamus is embedded in both the whole-brain connectome and, more specifically, the canonical reading network. Of interest was its association with reading subskills in children with and without RD. We found that efficiency measures of the left thalamus within a subnetwork composite of reading network regions and connectomic hubs were associated with RAN ability, which captures attentional control and visual-articulatory coordination demands of fluent reading. Moreover, children with better phonemic decoding abilities exhibited higher transmission costs of reading network pathways that pass through the thalamus. Both these findings were replicated in an independent sample of children, suggesting these are robust associations that represent fundamental differences in the white matter topography of individuals with a wide range of reading abilities.

## AUTHOR CONTRIBUTIONS

Chenglin Lou: Data curation; Formal analysis; Writing – original draft; Writing – review & editing. Alexandra M. Cross: Data curation; Funding acquisition; Writing – review & editing. Lien Peters: Data curation; Funding acquisition; Writing – review & editing. Daniel Ansari: Funding acquisition; Resources; Writing – review & editing. Marc F. Joanisse: Supervision; Writing – review & editing.

## FUNDING INFORMATION

Chenglin Lou, Western Graduate Research Scholarship. Marc F. Joanisse, NSERC Discovery Grant. Daniel Ansari, Canada First Research Excellence Fund to BrainsCAN. Daniel Ansari, Klaus J. Jacobs Foundation. Lien Peters, Brain and Mind Institute Post-doctoral fellowship. Lien Peters, Children’s Health Research Institute (CHRI) Trainee Award.

## CODE AND DATA AVAILABILITY

Code in support of this study is available on https://github.com/chenglinlou/reading_wm_connectome_thalamus. Our ethics approval does not permit sharing raw data in a public repository. However, our data for the primary analyses are available to researchers upon reasonable request, subject to approval from our local research ethics authority. The validation dataset is available on https://openneuro.org/datasets/ds001894/versions/1.4.2/metadata.

## TECHNICAL TERMS

Dyslexia: A learning disability that specifically affects reading; generally understood to affect learning to associate speech sounds to the written language.
Diffusion tensor imaging (DTI): A modality of MRI capturing microstructure features of white matter via water molecule diffusion.
White matter: A bundle of myelinated axons connecting various gray matter regions in the brain.
Connectome: A comprehensive map of structural connections between each pair of brain regions.
Hub: A node in a graph that has an more links to other nodes than average, and supports efficient long-distance communication.
Network topology: The arrangement of nodes and edges of a network.
Routing strategy: A model describing how information traverses a network in order to connect any two nodes in a graph.
Rapid automatized naming (RAN): A task measuring how quickly a person can name arrays of visual items such as letters, digits, objects, or colors.
Phonemic decoding: Recognizing a visual word by connecting visual letters to speech sounds.
